# Transversal Arch Clamping for Complete Resection of Aneurysms of the Distal Ascending Aorta without Open Anastomosis

**DOI:** 10.3390/jcm11102698

**Published:** 2022-05-10

**Authors:** Andreas Rukosujew, Arash Motekallemi, Konrad Wisniewski, Raluca Weber, Fernando De Torres-Alba, Abdulhakim Ibrahim, Raphael Weiss, Sven Martens, Angelo Maria Dell’Aquila

**Affiliations:** 1 Department of Cardiothoracic Surgery, University Hospital Muenster, 48149 Münster, Germany; arash.motekallemi@ukmuenster.de (A.M.); konrad.wisniewski@ukmuenster.de (K.W.); raluca.weber@ukmuenster.de (R.W.); sven.martens@ukmuenster.de (S.M.); angelo.dellaquila@ukmuenster.de (A.M.D.); 2 Department of Cardiology, University Hospital Muenster, 48149 Münster, Germany; fernando.detorresalba@ukmuenster.de; 3 Department of Vascular and Endovascular Surgery, University Hospital Münster, 48149 Münster, Germany; abdulhakim.ibrahim@ukmuenster.de; 4 Department of Anesthesiology, Intensive Care Medicine and Pain Therapy, University Hospital Münster, 48149 Münster, Germany; raphael.weiss@ukmuenster.de

**Keywords:** ascending aorta aneurysm, aortic replacement, technique of distal anastomosis

## Abstract

Background: The extent of aortic replacement for aneurysms of the distal ascending aorta remains controversial and opinions vary between standard cross-clamp resection and open hemiarch anastomosis in circulatory arrest and selective cerebral perfusion. As the deleterious effects of extended circulatory arrest are well-known, borderline indication for distal ascending aorta aneurysm repair must be outweighed against the potential risk of complications related to the open anastomosis. In the present study, we describe our own approach consisting of “transversal arch clamping” for exhaustive resection of aneurysms of the distal ascending aorta without open anastomosis and we present the postoperative outcomes. Methods: Between May 2017 and December 2019, 35 patients with aneurysm of the ascending aorta (20 male, 15 female) underwent replacement with repair of the lesser curvature without circulatory arrest. Pre-operative, intraoperative, and postoperative clinical outcomes were retrospectively withdrawn from our institutional database and analyzed. Results: Maximal diameter of distal ascending aorta was 47.5 mm. Patient median age was 66 years (IQR 14) (range 42–86). Preoperative logistic median EuroSCORE II was 17% (IQR 11.3). Median duration of cardiopulmonary bypass and cardiac arrest were 137 (IQR 64) and 93 (IQR 59) min, respectively. In-hospital and 30-day mortality were 0%. There were no cases with acute low output syndrome, surgical re-exploration for bleeding, kidney injury requiring dialysis, or wound infection. Disabling stroke was observed in one patient (2.9%). There was one case of major ventricular arrhythmia (2.9%). Conclusions: Our institutional experience suggests that this novel technique is safe and feasible. It facilitates complete resection of the aortic ascending aneurysm avoiding circulatory arrest, antegrade cerebral perfusion, additional peripheral cannulation, and all related complications.

## 1. Introduction

Regarding current evidence, the extent of aortic replacement in borderline aneurysms of the distal ascending aorta remains controversial and opinions vary between standard cross-clamp aortic resection and open hemiarch anastomosis in hypothermic circulatory arrest [1,2,3]. During the so-called “conventional” ascending aorta replacement, approximately 2 cm of the distal aorta ascendens may remain unresected without open anastomosis technique. This could lead to a certain degree of diameter mismatch between graft and proximal arch. As a consequence, the remaining aneurysmatic tissue may predispose patients to a further arch dilatation with aneurysm formation on the long run. On the other hand, “open” distal anastomosis and hemiarch reconstruction in hypothermic circulatory arrest (HCA) allows a more complete resection of aneurysmatic tissue [3,4,5]. However, the use of HCA results in prolonged extracorporeal circulation with potential end organ ischemia. In addition, this procedure requires a peripheral arterial cannulation that can potentially cause further related complications.

There are several surgical techniques concerning distal anastomosis for the resection of the ascending aorta aneurysms with or without HCA [1,6,7]. At our institution, we developed a new approach of transversal arch clamping with closed distal anastomosis for avoiding circulatory arrest, antegrade cerebral perfusion, and additional peripheral cannulation while allowing a more complete resection of aneurysmatic tissue.

In this study, we present our technique and discuss our institutional experience and outcomes in patients with borderline indication for aneurysms of the distal ascending aorta. Moreover, we retrospectively analyzed aortic diameter size of the resected aneurysmatic aorta in order to identify a potential reference value for the application of our institutional method.

## 2. Patients and Methods

Local ethics committee approval was granted for the collection of patient data as well as follow up (approval number 2020-076-f-S). The present study includes 35 patients between May 2017 and December 2019 who underwent an elective replacement of an ascending aorta aneurysm with our institutional method of transversal arch clamping. The preoperative workup included either computed tomography angiography (CTA), echocardiography, or coronary angiography (in patients older than 50 years). The indication for surgical aortic replacement was according to the 2014 ESC Guidelines [8]. The decision whether patients were suitable for “transversal arch clamping” was made intraoperatively, based on surgical assessment and the extent of aneurysm reaching into the aortic arch. All baseline data of patients, including ascending aorta diameter, aortic valve characteristics, and major comorbidities are presented in Table 1.

IBM SPSS Statistics for Windows, Version 22 (IBM Corp, Armonk, NY, USA) was used for statistical analysis. A Kolmogorow–Smirnow test with Lilliefors correction was applied across the data and revealed a normal distribution of data regarding aortic diameter (K = 0.19635, *p* = 0.05217). Median was used presenting the variables.

### 2.1. Surgical Technique

**Figure 1:**
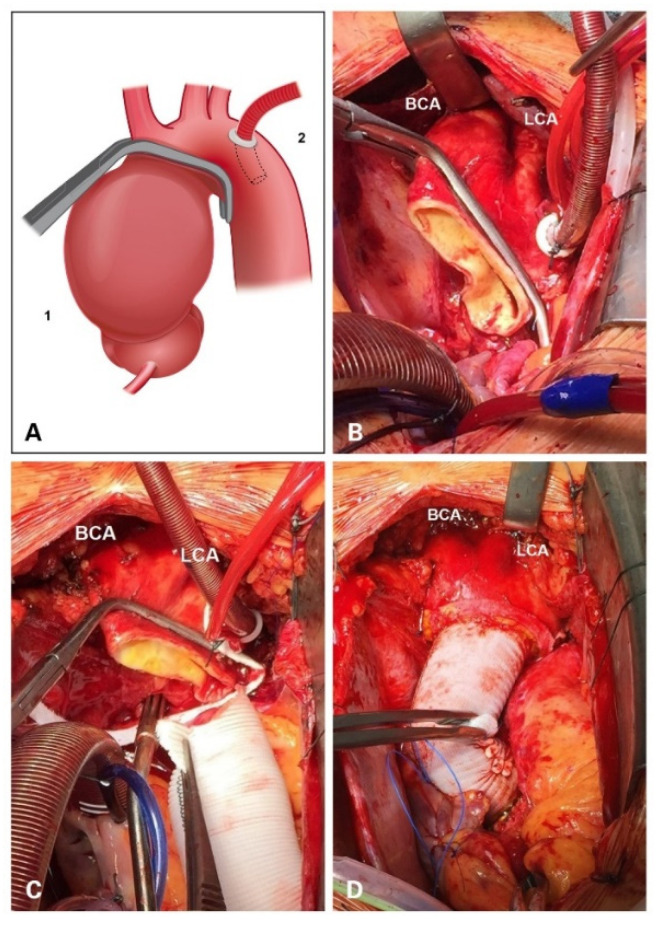
Stepwise approach of our institutional method of transversal arch clamping. (**A**) A schematic picture of the Satinsky clamp placement in relation to ascending aneurysm (1) and the position of the aortic cannula (2). (**B**) Intraoperative view showing complete resection of ascending aortic aneurysm. (**C**) Display of the Satinsky clamp for the “transversal arch clamping”. (**D**) A picture after the completion of ascending aorta replacement and repair of the lesser curvature of the aortic arch. BCA: brachiocephalic artery; LCA: left carotid artery.

NIRS control with left radial and femoral pressure monitoring was applied during all procedures. Cardiopulmonary bypass was established using arterial cannulation of the distal aortic arch directly below the left subclavian artery and venous cannulation of the right atrium. In most cases, Seldinger technique with echocardiographic control of the wire position in the aorta descendens was used for arterial cannulation. In our opinion based on our institutional experience, in this way, the cannula can be safely introduced at an acute angle in the descending aorta facilitating cannulation. Without Seldinger technique there is the risk of potential aortic dissection due to tangential introduction of the cannula. Myocardial protection is achieved either by retrograde cold blood cardioplegia in case of full median sternotomy or, in case of L-shaped partial upper sternotomy, initially anterograde through the aortic wall followed by selective cannulation of coronary ostia. Figure 1 demonstrates our institutional approach step by step.

First, a conventional clamping is performed proximal to the brachiocephalic artery (BCA) (Figure 1A). Second, in cardiac arrest, aneurysmatic tissue is resected right below the clamp (at the level of the BCA) and at the sinotubular junction (STJ). Once the ascending aorta has been trimmed, the aortic arch should be mobilized dorsally by separating the aorta from the periaortic tissue. In this regard, it is of utmost importance to extend the distal preparation exposing zone 3 of the aorta. At this point, the Satinsky clamp can be safely placed in order to expose the lesser curvature for removal. This maneuver enables complete mobilization of the aortic arch under visual control. Third, a Satinsky clamp is then (under low-flow cardiopulmonary bypass) placed distal to the first clamp at the outflow of the brachiocephalic artery and transversal to the aortic arch allowing maximal removal of the lesser curvature (Figure 1B,C). Prosthesis size is determined according to the diameter of the sinotubular junction in case of a supracoronary replacement or to conduit size when aortic root is replaced. The selected prosthesis is trimmed and tailored for distal anastomosis leaving a prosthesis bevel for accommodation to the lesser curvature. After proximal arch resection the distal anastomosis is carried out by means of continuous 4-0 polypropylene suture using a periaortic Teflon felt strip. The proximal anastomosis was then tailored according to the necessity of concomitant procedures (i.e., composite graft implantation, aortic root reconstruction). BioGlue^®^ (CryoLife, Inc., Kennesaw, GA, USA) was applied for sealing of the suture line (Figure 1D).

### 2.2. Retrospective Biplanar Measurement

In order to identify a potential reference value for the application of our institutional method regarding suitable aneurysmatic diameter at the different levels of the ascending aorta we retrospectively performed biplanar measurement. CT angiography scans were reconstructed automatically using Aquarius iNtuition (TeraRecon Inc., Foster City, CA, USA).

**Figure 2:**
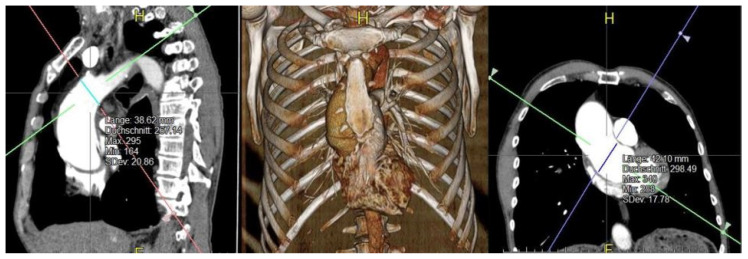
Exemplary biplanar measurement with automated 3D reconstruction using Aquarius iNtuition.

Based on ECG-gated computed tomography angiograms (CTAs) biplanar measurements at the level of the sinotubular junction (STJ) and immediately proximal to the origin of the brachiocephalic artery (BCA), the supposed position of the clamp, have been performed. Exemplary biplanar measurement with automated 3D reconstruction is displayed in Figure 2. According to the data, a suggested reference value for suitable diameter size of the respected levels of aneurysmatic aorta for the application of our institutional method of transversal arch clamping was evaluated.

## 3. Results

Patient median age was 66 years (IQR 14) (range 42–86). Patient demographics and concomitant procedures are summarized in Table 1. The median diameter of the ascending aorta was 57 mm (IQR 7) (range 50–72 mm). Half of the patients (51%) had a bicuspid aortic valve. Preoperative EuroSCORE II was 8.7 (IQR 2.5).

The median duration of surgery, CPB time and cross clamp time were 232, 137, and 93 min, respectively. Mild hypothermia was applied in all cases with average nadir temperature about 32 °C. Concomitant CABG procedure took place in 10 (28.6%) patients. Additional Bentall operation, partial Yacoub procedure, and Wheat procedure were performed in 14 (40.0%), 5 (14.3%), and 3 (8.6%) patients of the study group, respectively. Minimally invasive approach through L-shaped partial upper sternotomy was performed in 8 (22.9%) patients. Intraoperative data and concomitant procedures are summarized in Table 2.

The postoperative results are shown in Table 3. There were neither in-hospital nor 30-day mortality cases. The length of ICU and IMC stay was 2.8 (IQR 2.5) days, the median mechanical ventilation time lasted 9.4 (IQR 3) h. The mean amount of blood loss was 724 (IQR 320) mL and none of the patients required a re-exploration for revision. One patient (2.9%) had a postoperative stroke with residual hemiparesis at discharge. Delirium requiring drug treatment was reported in nine patients (27.7%) There was no postoperative kidney injury requiring dialysis. In one patient with postoperative creatinine value of 2.6 mg/dL the renal function was restored due to medical treatment and volume management. Deep wound infections were not observed. In 19 out of 35 patients, CTA based, biplanar measurements of the sinotubular junction (STJ) and the base of the brachiocephalic artery (the designated clamping site) were analyzed retrospectively in order to objectify the surgeon’s “instinct” and identify a potential reference size (regarding suitable diameter) for the application of this technique.

The missing CTAs are due to the following reasons: In ten patients with bicuspid valve and dilatated aorta echocardiography was used for indication. In two patients, indication was based on aortography due to concomitant CABG procedure. In four patients the decision was made intraoperatively without additional preoperative imaging. Using Aquarius iNtuition^®^ for biplanar measurement the median diameter of the STJ and BCA origin (designated clamping site) were 45.7 mm (range: 24.5–57 mm) and 40.9 mm (range: 35.7–47.5 mm), respectively.

## 4. Discussion

To date, there is no consensus regarding the optimal surgical approach for borderline aneurysms of the distal ascending aorta. Although open hemiarch anastomosis requiring initial peripheral cannulation and HCA is necessary for the treatment of type A aortic dissection, the same approach seems exaggerated for the purpose of extensive repair of borderline aneurysms of the distal ascending aorta, even if parts of the aortic arch are involved. However, several retrospective studies [9,10] have shown that the open hemiarch approach is a similarly safe method and does not increase the risk for cardiac, neurological, pulmonary, or hemorrhagic complications in the immediate postoperative period. On the other hand, no prospective studies are available. We know from our own experience that aortic surgery with HCA is not an “easy walk” and contains a potential risk of coagulation disorder with postoperative bleeding as well as potential cerebral injury as a result of air embolism, insufficient perfusion, or other potential complications like ischemia of the abdominal organs or extremities. Furthermore, the extrathoracic arterial cannulation itself via right axillary artery and/or femoral artery carries the risk for associated complications such as brachial plexus injury, ischemia, bleeding, and lymphatic fistula.

In our opinion, the above-mentioned potential risks for the treatment of borderline aneurysms of the distal ascending aorta do not justify a rigid application for aneurysms of the distal ascending aorta. Rather, a tailored approach seems to be indicated as it enables to maximize resection of aneurysmatic tissue—even in cases with aortic arch involvement.

Our institutional approach claims to achieve both, balancing exhaustive resection of aneurysmatic tissue while preventing additional damage. In our setting, despite the double clamping of the aorta (first, in a conventional manner and then transversally—see Figure 1), the surgeon can achieve a more exhaustive resection and thus reduce the associated risks of undissected aneurysmatic tissue. This is reached through the maneuver of clamp replacement under visual control through mobilization of the dorsal wall of the aortic arch. This allows the application of the Satinsky clamp without multiple attempts and thus avoids possible iatrogenic aortic wall injury.

In our institutional experience, this technique can be applied safely in the majority of patients with borderline aneurysms. However, one must consider that any multiple replacements of the clamp could lead to potential plaque loosing or rupture. It is self-explanatory that this should be avoided in patients with sclerotic distal ascending aortas or visible (via sonography) or manual palpation at the intended clamping site.

In our patient population, there was no bleeding at the distal anastomosis region as a result of the suture penetration due to aortic tension during clamping. The application of the transversal arch clamping generally did not result in a deviation from the initial surgical plan. In our opinion, this is due to the accurate preoperative planning. Moreover, our outcomes suggest that this method does not increase the risk for additional neurological deficits, strokes, or an increase in cardiac or non-cardiac associated death.

We also believe that our technique with formation of a “prosthesis bevel” for repair of the lesser curvature of the aortic arch stabilizes the aortic arch and thus prevents the formation of new aneurysms, even if performed in an open manner.

Another benefit of this approach might be the prevention of hypothermic circulatory arrest enabling even the “lesser experienced” surgeon to reach maximized resection of all aneurysmatic tissue in even more complex cases of borderline aneurysms of the distal ascending aorta. This enables low volume centers to reach better outcomes. We have identified some limitations of our study, which are mainly expressed by the retrospective design and the lack of a control group. We aimed to reduce the subjective assessment of the surgeon by identifying a potential reference value for the application of our institutional method regarding suitable aneurysmatic diameter at the different levels of the ascending aorta through biplanar measurement.

Our data suggests that up to an aortic diameter of 47.5 mm at the BCA origin designated clamping site our method of transversal arch clamping can safely be applied. Routinely implemented biplanar measurement might become a standardized approach for the assessment of aneurysmatic aneurysms to identify suitable candidates for this approach.

## 5. Conclusions

Transversal arch clamping of aneurysms of the distal ascending aorta (reaching into the aortic arch) seems to be a safe and feasible method in order to achieve maximized resection of aneurysmatic tissue. Waiving the disadvantages of hypothermic circulatory arrest with antegrade cerebral perfusion and potential risks of peripheral cannulation may qualify this approach to become a standard approach in low volume centers and for less experienced surgeons.

## Figures and Tables

**Table 1:** Demographic patient data.

Preoperative Variables	Median/N	IQR/%
Age (years)	66	14
Sex (female)	15	43%
BMI	29	5
History of stroke	14	40%
Diabetes mellitus	3	8.6%
Dyslipidemia	13	37.1%
Arterial hypertension	28	80%
Peripheral vascular disease	1	2.9%
Cerebrovascular disease	2	5.7%
Abdominal aortic aneurysm	1	2.9%
COPD	7	20%
Preoperative history of stroke	3	8.6%
NYHA	1.8	1
Creatinine peak (mg/dL)	1.0	0.3
Hemoglobin (g/dL)	13.9	2.6
Atrial fibrillation	7	20%
Aortic diameter (mm)	57	7
Bicuspid aortic valve	18	51%
Redo	2	5.7%
EuroSCORE II	8.7	2.5

**Table 2:** Intraoperative data and concomitant procedures.

Operative Variables	Median/N	IQR/%
Duration of surgery (min.)	232	99
CPB time (min.)	137	64
Cross clamp time (min.)	93	59
Nadir temperature (min.)	32	1
Concomitant CABG	10	28.6%
Bentall operation	14	40.0%
Partial Yacoub procedure	5	14.3%
Wheat procedure	2	5.7%
Partial upper sternotomy	8	22.9%

**Table 3:** Postoperative outcomes.

Postoperative Variables	Median/N	IQR/%
Hospital stay (d)	10	2.5
In-hospital mortality	0	0%
30-day mortality	0	0%
Length of ICU/IMC stay (d)	2.8	2.5
Duration of mechanical ventilation (h)	9.4	3
Tracheostomy	0	0%
Low output syndrome	0	0%
Surgical re-exploration for bleeding	0	0%
Drainage loss (mL)	724	320
CPR	1	2.9%
Disabling stroke	1	2.9%
Delirium	9	27.7%
Dialysis	0	0%
Creatinine peak mg/dL	0.9	0.4
Wound/sternal infection	0	0%
NYHA	1.6	1

## Data Availability

Raw data were generated at the Department of Cardiothoracic Surgery, University Hospital Muenster, Muenster, Germany. Derived data supporting the findings of this study are available from the corresponding author Andreas Rukosujew on request.
